# Allogeneic transplantation for high-risk chronic lymphocytic leukemia—a summary of a 16-year experience

**DOI:** 10.1007/s00277-019-03679-x

**Published:** 2019-03-27

**Authors:** Grzegorz Helbig, Adrianna Spałek, Agata Wieczorkiewicz-Kabut, Mirosław Markiewicz, Małgorzata Kopera, Patrycja Zielińska, Krzysztof Woźniczka, Anna Kopińska, Iwona Grygoruk-Wiśniowska, Anna Koclęga

**Affiliations:** School of Medicine in Katowice, Department of Hematology and Bone Marrow Transplantation, Medical University of Silesia, Dąbrowski street 25, 40-032 Katowice, Poland

**Keywords:** Chronic lymphocytic leukemia, Allogeneic stem cell transplantation, Outcome, Reduced intensity conditioning, Myeloablative conditioning, Survival, Mortality

## Abstract

In the pathway inhibitor era, the number of allogeneic stem cell transplantation (ASCT) for chronic lymphocytic leukemia (CLL) continues to decrease and this approach should be offered only after careful risk-benefit assessment. Nevertheless, ASCT still remains only curative therapeutic modality for CLL, especially in countries with limited access to novel agents. Thirty patients with CLL at median age of 42 years at diagnosis (range 29–64) underwent ASCT between years 2002 and 2018. Thirteen patients were transplanted in complete remission (CR), ten patients achieved partial response (PR), and seven had stable disease. The median time from diagnosis to transplant was 4 years (range 0.5–12). Twenty-three patients received HLA-matched related donor stem cell grafts, and seven patients received either matched unrelated donor or HLA-mismatched grafts. Reduced intensity conditioning (RIC) and myeloablative regimen (MAC) were used in 24 and 6 patients, respectively. Mortality to day + 100 after transplant was 16% (8% for RIC only). Acute and chronic graft versus host disease (GVHD) developed in 40% and 63% of patients, respectively. Fifteen patients relapsed or progressed after transplant. Thirteen patients (43%) are alive at last follow-up and 10 (77%) remain in clinical CR. Median follow-up for survivors was 6.8 years (range 0.4–15.2). Three-year progression-free and overall survivals were 56% and 60%, respectively. These outcomes were better for patients who received RIC conditioning: 64% and 72%, respectively. CR at transplant was found to have favorable impact on post-allograft survival. RIC should be preferred over MAC. ASCT may remain a valuable option for some CLL patients.

## Introduction

Allogeneic stem cell transplantation (ASCT) remains the only curative treatment for patients with chronic lymphocytic leukemia (CLL) despite the wide availability of novel targeted agents. As CLL predominates in elderly, only a small proportion of patients are eligible for allogeneic transplantation. Moreover, in the era of oral, well-tolerated, and effective drugs, decision on transplantation must weigh up the risk and benefits. Every candidate for ASCT should be aware of potential life-threatening complications and the final decision should take into account the expectations of the patient [1]. The main task of treating physician is to identify “high-risk” CLL patients who should be offered ASCT. More than a decade ago, the criteria of poor prognosis CLL were proposed and then widely accepted. Briefly, they included CLL patients who (1) had deletion 17p or TP53 mutation with treatment indication, (2) were refractory to (or had early < 12 months relapse) purine analog-based therapy, and (3) had relapse < 24 months after purine analog combinations or autologous stem cell transplantation [2]. However, the introduction of chemo-immunotherapy and B-cell receptor/Bcl-2 inhibitors has changed our therapeutic approach to patients with CLL and eligibility criteria for ASCT. Taking all this into account, a new prognostic model influencing the treatment decision has been recently proposed; however, it overlaps with earlier ones. The one exception is that chemotherapy was replaced by chemo-immunotherapy [3]. Nevertheless, it is clear now that the targeted therapy dramatically decreased the number of ASCT in CLL patients. According to the recent report of European Society for Blood and Marrow Transplant (EBMT), ASCT for CLL constitutes 2% of all allogeneic transplants with total number of 275 in 2016 [4]. However, it seems that ASCT still remains a therapeutic option, especially for patients who fail, are intolerant, or do not have access to novel agents. The latter issue concerns polish patients with CLL for whom the pathway inhibitors are not widely available. Nowadays, ~ 4 allogeneic transplants for CLL were performed yearly in our center.

Here, we present the results of our retrospective analysis of 30 allogeneic transplants for CLL performed in the last 16 years.

## Material and methods

The study patients were identified through the use of our institutional database of medical records. The diagnosis of CLL and response criteria to therapy were based on National Cancer Institute-Sponsored Working Group criteria (NCI/WG) [5, 6]. The eligible patients met at least one EBMT criterion for high-risk CLL [2, 7]. Clinical response was based on results of peripheral blood, bone marrow, and imaging studies (ultrasound and/or CT) and assessed before transplantation and every 3–6 months after transplantation. In the case of complete or partial responses (CR/PR), immunophenotypic studies measuring minimal residual disease (MRD) were recommended as described elsewhere [8]. Flow cytometry analysis of lymphoid cells in peripheral blood was performed at diagnosis. MRD was measured on day + 30 after transplant and then in patients who achieved clinical CR/PR. Of note is that data are missing in some patients on different stages of measurements. Data on cytogenetics by fluorescence in situ hybridization (FISH) were not available in 60% of study patients, the remaining 40% were categorized into four risk groups as it was proposed by Rossi et al. [9]. Chimerism of unseparated blood leukocytes was assessed by short tandem repeat polymerase chain reaction. Not all data were available due to the retrospective nature of the study. All patients provided an informed consent in accordance with the Declaration of Helsinki.

## Statistics

Nonparametric comparisons of group means were performed by using the Mann-Whitney *U* test. Proportions were compared by Fisher exact test. Non-relapse mortality (NRM) was defined as all deaths before clinical progression or disease recurrence. The distribution for overall survival (OS) and progression-free survival (PFS) was estimated using the method of Kaplan and Meier and compared using the log-rank test. A *p* value less than .05 was considered significant. Proportional hazard models (Cox regression) were fitted to investigate effects of prognostic factors for OS. The following factors were entered into model (1) patient-related: age, Rai’s stage, Binet’s stage, clinical and MRD disease status at transplant and (2) transplant-related: donor age, donor type, type of conditioning, stem cell source, date of transplant. All computations were performed with StatSoft Poland analysis software (version 10.0).

## Results

### Patient characteristics

Thirty patients (20 males and 10 females) with CLL at median age of 42 years at diagnosis (range 29–64) underwent ASCT between 2002 and 2018. Most frequent pre-transplant chemotherapy included fludarabine and cyclophosphamide ± rituximab (FC ± R; 83%) and cyclophosphamide, vincristine, prednisone ± doxorubicin ± rituximab (CHOP/CVP ± R; 40%). Two patients underwent prior autologous stem cell transplantation. Sixteen patients (53%) had received only a single treatment line (most frequently FC ± R regimen) before transplant; however, none of them was transplanted in CR. These patients had PR or stable disease (SD) at transplant. CR was demonstrated after at least two treatment lines. Clinical status at transplant was as follows: CR in 13 patients; 4 of them demonstrated also MRD negativity in peripheral blood. PR was found in ten patients whereas SD occurred in seven cases. MRD measurement in peripheral blood was found negative in five patients (including four with CR and one with PR). Sixty percent of patients demonstrated MRD positivity at transplant. Median time from diagnosis to transplant was 4 years (range 0.5–12). Patients’ characteristics are shown in Table 1.

**Table 1:** Patients characteristics

Parameter	*n* = 30 (%)
Gender (female/male)	10/20
Age at diagnosis (median, range); years	41.5 (29–64)
Rai’s stage at diagnosis	
0	6 (20)
I	17 (56)
II	2 (7)
III	5 (17)
IV	0 (0)
Binet’s stage at diagnosis	
A	10 (33)
B	15 (50)
C	5 (17)
Number of treatment lines before transplant (median, range)	1 (1–6)
Therapy before transplant	
FC ± R	25 (83)
CHOP + R	7 (23)
CVP ± R	5 (17)
Chlorambucil ± prednisone	4 (13)
F + alemtuzumab	3 (10)
R-DHAP	2 (7)
RB	2 (7)
Other	3 (10)
Autologous transplantation	2 (7)
Prior radiotherapy	15 (50)
Response at transplant	
CR	13 (43)
PR	10 (33)
SD	7 (24)
MRD before transplant	
Negative	5 (17)
Positive	18 (60)
Missing	7 (23)
Median time from diagnosis to transplant (median, range); years	4.1 (0.5–11.9)

*CR* complete response, *PR* partial response, *SD* stable disease, *MRD* minimal residual disease, *FISH* fluorescence in situ hybridization, *DHAP* dexamethasone, cisplatin, cytarabine, *B* bendamustine

### Transplant data

#### Baseline characteristics of transplanted patients

Median recipient age was 46.5 years (range 31–66). Median donor age was 43 years (range 19–72); however, unrelated donors were significantly younger than siblings (30 years vs 45 years; *p* = 0.001). Sixteen patients were transplanted before year 2010. Twenty-three patients received HLA-matched related donor stem cell grafts, and seven patients received either matched unrelated donor (*n* = 5) or HLA-mismatched grafts (*n* = 2). Peripheral blood was a source of stem cells for 24 patients, and 6 patients received stem cells from bone marrow. In total, 24 patients received reduced intensity conditioning (RIC) and 16 of them (66%) were additionally given monoclonal antibodies either rituximab or alemtuzumab. Myeloablative conditioning (MAC) consisting of cyclophosphamide and total body irradiation was used in six patients. Anti-thymocyte globulin (ATG) was administered in seven patients who received a graft from unrelated donors. Graft versus host disease (GHVD) prophylaxis included cyclosporine (*n* = 28) or tacrolimus (*n* = 2) with methotrexate.

#### Outcome of transplanted patients

There were two primary graft failures (PGF) and these patients required second ASCT. Two patients died within the first 30 days after transplant due to infectious complications. Three other infection-related deaths were observed to day + 100 after transplant. Mortality to day + 100 was 16%. Three deaths (50%) were observed in MAC group and two deaths occurred in patients transplanted with RIC (8%).

Thirteen out of 29 evaluated patients (44%) had negative MRD on peripheral blood on day + 30. Eleven patients demonstrated a full donor chimerism on the day of discharge and 10 patients had mixed chimerism. Data were not available for six patients. Two patients had PGF. Donor lymphocytes for MRD positivity after transplant were infused in eight patients; however, 60% of them progressed.

Acute and chronic graft versus host disease (GVHD) developed in 40% and 63% of patients, respectively. Acute GVHD grade III/IV was present in only two patients. Eighteen patients had limited and one extensive chronic GVHD. There were no differences in acute GVHD, chronic GVHD, and post-transplant infection rates between following groups: ATG vs non-ATG, RIC vs MAC, and BM vs PB.

Patients who received RIC regimen when compared to MAC had comparable CR rates (62% vs 33%; *p* = 0.5), but OS at 3 years was significantly higher in the former group: 72% vs 16%; *p* = 0.01. In total, 11 patients died in RIC and all 6 patients in MAC group. Disease progression was the main cause of death in 54% of RIC patients and 33% of MAC.

Ten patients demonstrated complications during early post-transplant hospitalization. There were two cases of fungal infections (pulmonary aspergillosis), one of these patients received conditioning with alemtuzumab. The other complications were moderate and included bacterial pneumonia (*n* = 1), fever of unknown origin (*n* = 3), cystitis (*n* = 1), *Clostridium difficile*-negative diarrhea (*n* = 3), and mucositis grade 3 (*n* = 4). One patient developed deep vein thrombosis.

Post-allograft CR rate was 50% including those with CR before procedure. Fifteen patients relapsed or progressed. The median time to relapse/progression was 2 years (range 0.9–4.1). Four patients required second transplant as a consequence of graft failure (*n* = 2) or disease progression (*n* = 2). At the last follow-up, 17 patients died. The main causes of death included disease progression (*n* = 6), infectious complications (*n* = 6), secondary graft failures (*n* = 4), and extensive chronic GVHD (*n* = 1). In total, there were five deaths within the first 2 years after transplant, eight patients expired between 2 and 5 years, and one patient died > 5 years after procedure. NRM at 3 years was 36% for entire cohort and only 12% for RIC.

Thirteen patients (43%) are alive at the last contact and 12 of them were found to have full donor chimerism. Ten patients remain in clinical CR. Median time from diagnosis to last contact reached 8.3 years (range 1.8–19.0). Median follow-up for survivors was 6.8 years (range 0.4–15.2). Transplant data are summarized in Table 2.

**Table 2:** Transplant data

Parameter	*N* = 30 (%)
Age of recipient (median, range); years	46.5 (31–66)
Age of donor (median, range); years	43 (19–72)
Related	45 (19–72)
Unrelated	30 (22–39)
Type of donor	
Related	23 (77)
HLA 10/10 unrelated	5 (17)
HLA 9/10 unrelated	2 (6)
Date of transplant	
< 2010	16 (53)
≥ 2010	14 (47)
Conditioning	
FLU/MEL + ALEM	12 (40)
BU/TREO/MEL + FLU	8 (30)
CTX + TBI	6 (20)
FLU/MEL + R	2 (5)
FLU/BENDA + R	2 (5)
Number of transplanted CD34-positive cells (× 10^6^/kg); median, range	4.3 (0.86–13.6)
ANC > 0.5 (× 10^9^/L); median, range	19 (13–119)
PLT > 20 (× 10^9^/L); median, range	17 (10–103)
No engraftment	2 (7)
Acute GVHD	12 (40)
Chronic GVHD	19 (63)
Life-threatening infectious complications to day + 30	2 (7)
Relapse/progression	15 (50)
Alive at last contact	13 (43)
Median follow-up (years); median, range	3 (0.1–15.2)

*FLU* fludarabine, *MEL* melphalan, *ALEM* alemtuzumab, *BU* busulphan, *TREO* treosulphan, *CTX* cyclophosphamide, *TBI* total body irradiation, *R* rituximab, *BENDA* bendamustine, *ANC* absolute neutrophil count, *PLT* platelets, *GHVD* fradt versus host disease

**Fig. 1:**
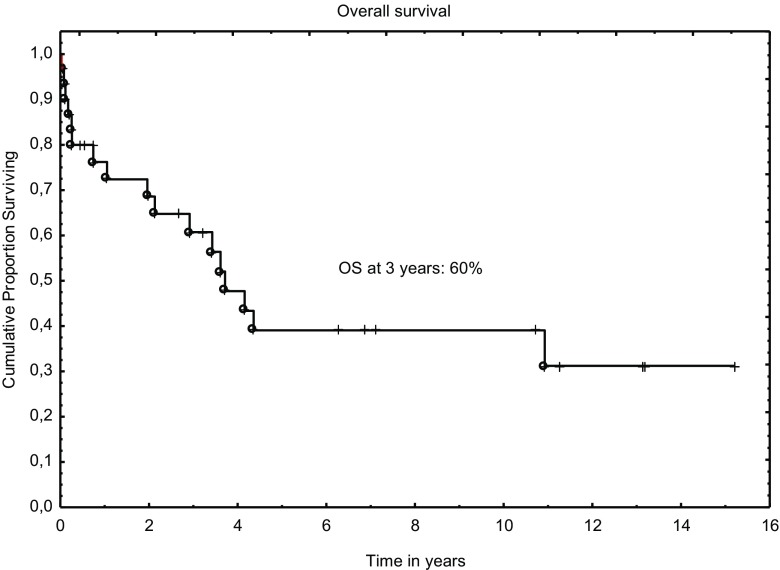
Overall survival after allograft for CLL

**Fig. 2:**
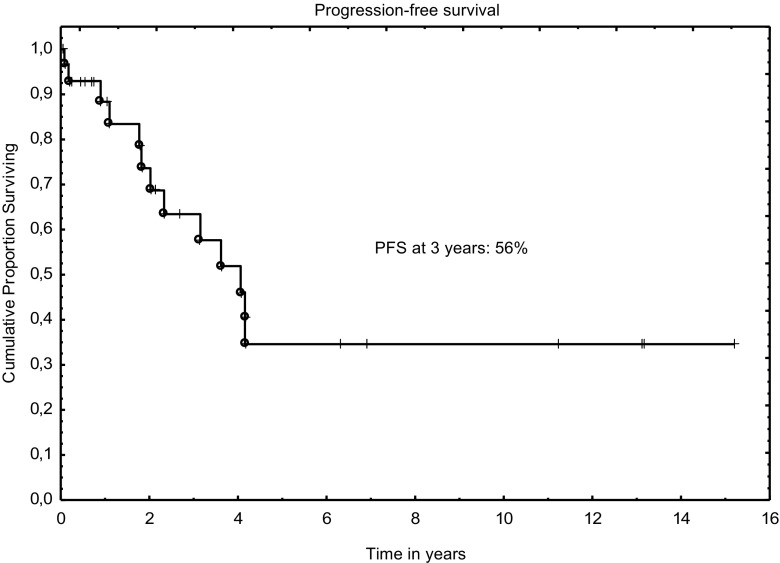
Progression-free survival after allograft for CLL

**Fig. 3:**
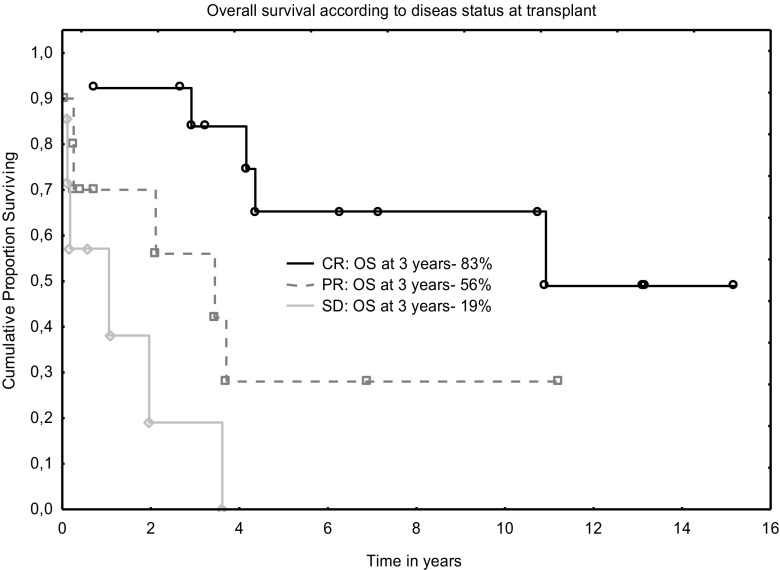
Overall survival for CLL after allograft according to disease status

Three-year progression-free and overall survivals were 56% and 60%, respectively (Figs. 1 and 2). The outcomes were better when analyzed only for RIC: 64% and 72% for PFS and OS, respectively. In univariable analysis, age at diagnosis, Binet’s stage, type of conditioning, clinical and MRD disease status before transplant were found to influence OS; however, only status of clinical response remained significant in multivariable analysis (Table 3 and Fig. 3).

**Table 3:** Univariate and multivariate analysis of risk factors for overall survival

Risk factor	HR (95%CI)	*P* value	HR (95%CI)	*P* value
Age	0.92 (0.86–1.07)	0.09	–	–
Binet stage at diagnosis	1.78 (0.9–3.3)	0.07	–	–
Disease status before transplant	3.12 (1.5–6.1)	0.001	2.07 (1.1–3.8)	0.02
MRD at treatment completion	3.26 (1.4–7.5)	0.006	–	–
Type of conditioning	2.1 (1.2–3.6)	0.008	–	–

## Discussion

Allogeneic stem cell transplantation remains the only curative therapeutic option for high-risk patients with CLL, but high efficacy of novel oral agents has changed our view who is a suitable candidate for transplantation [7]. The long-term effect of pathway inhibitors on post-transplant safety and efficacy requires to be elucidated. Only single retrospective study reporting data on BCR-inhibitor-ibrutinib for bridging to transplantation in CLL has been published so far. Forty-eight patients were included and 73% of them were ibrutinib-responsive at transplant. Twelve months relapse incidence (RI), progression-free/event-free (PFS/EFS), and overall survivals (OS) were 30%, 60%, and 72%, respectively. It was demonstrated that pre-transplant resistance to ibrutinib and poor performance status at transplant negatively influenced RI, PFS, and OS on multivariable analysis. Of note is that prior ibrutinib therapy did not affect the safety of subsequent transplantation [10]. Interestingly, RI was higher than previously reported for ibrutinib-naïve patients [11].

We performed 30 allogeneic transplants for CLL, but none of the patients received prior ibrutinib as its availability for polish patients was limited. In the era of BCR/bcl-2 inhibitors, it has been suggested that historically, poor-risk CLL (e.g., with TP53 mutation) should initially be treated with novel agents and transplanted when this therapy fails or unacceptable toxicities occur. In fact, the number of allogeneic transplants for CLL continues to decrease in Europe and USA in the recent years [3].

Herein, we present our results of allogeneic transplants which have been performed in the last 16 years. As there have been many drawbacks resulting from the retrospective nature of the study, the study shows how the pre-transplant conditioning and post-transplant care have altered over the years. When we divided our transplants into two groups depending on time of procedure, before and after year 2010, no significant difference in terms of early mortality, NRM, OS, and GVHD incidence has been demonstrated. Of note is that post-transplant infection rates were also comparable despite the fact that most patients received alemtuzumab before 2010 (data not shown).

Nowadays, RIC is commonly used before transplantation, and this is the case for most of our patients. The introduction of RIC makes the transplantation more accessible even in elderly and frail patients. However, only six of our patients were 60 years or older at transplant. The pooled data of post-transplant outcome according to conditioning intensity have demonstrated higher CR, PFS, and OS rates for RIC group when compared to MAC. Non-relapse mortality was lower in the former one. No difference between groups was found in terms of acute GVHD incidence, but chronic GVHD was more frequently observed in RIC patients. The latter was probably associated with G-CSF-mobilized stem cells from peripheral blood [12].

The retrospective results of RIC ASCT for poor-risk CLL were recently presented by Kharfan-Dabaja et al. [12]. Briefly, the number of transplanted patients varies between studies and ranged from 9 patients up to 180 (most studies included 30–40 patients) with median age of more than 50 years at transplant. The conditioning regimen varied as did donor type and stem cell source. Five-year OS ranged from 45 to 72%. Of note is that this systemic meta-analysis has many limitations resulting from data scarcity and incompleteness, long time period of collected data, and many other factors. Taken together, due to the diversity of studies, direct comparison between them is difficult. Nevertheless, our results were partly in line with pooled rates of post-allograft outcomes of retrospective studies [12]. Due to the small number of patients transplanted with MAC regimen, we were not able to compare our all outcomes with RIC, and the results should be treated with caution. Nevertheless, CR rates as well as acute and chronic GVHD rates did not differ. OS was significantly better in RIC and this finding was in line with data published elsewhere [12]. Worse OS in MAC patients when compared with RIC resulted from higher mortality rate in the former (all six patients died in MAC group). Transplant-related mortality for our cohort was 16%; however, it was only 8% when we analyzed patients transplanted with RIC. This is in accordance with data presented by others [13, 14].

It was demonstrated that RIC ASCT provides durable response in about 40% of CLL patients with mortality of less than 10% within the first 100 days after transplant. Median OS at 5 years is 50% [15, 16]. These results seem to be encouraging; however, the responses continue to decrease with time after transplant. The largest analysis of 2589 transplanted CLL patients was provided by the European Society for Blood and Marrow Transplantation (EBMT). It was clearly demonstrated that all outcomes (OS, NRM, EFS) decrease with long-term follow-up [16]. Nevertheless, plateau in PFS at 4 years (~ 35%) after allograft was demonstrated in our analysis.

The factors which may have an adverse impact on post-allograft EFS and OS were defined by German CLL Study Group and included alemtuzumab as a part of pre-transplant conditioning and active disease at transplant [15]. The latter was also found to negatively influence the survival in our cohort. Remission at transplant was found to be an important prognostic factor in other studies [11]. An important issue of ASCT for CLL patients is associated with post-allograft MRD monitoring. It was demonstrated that regular MRD assessment reduces relapse risk and provides longer EFS without increase of NRM. This beneficial effect was probably due to the use of preemptive DLI and shortening of immunosuppressive therapy [15]. The role of MRD was not an issue in our study due to the data incompleteness. DLI did not provide long-term disease control. It was demonstrated that immunologic effect acts poorly in CLL patients and fails to eradicate the disease [11].

Despite the high rate of CLL relapse or progression after ASCT, the patients still have a good prognosis and may benefit from other treatments, especially with the use of targeted agents [17]. In the BCR/Bcl-2 inhibitor era, the role of ASCT in CLL is markedly limited and this approach should be offered only after careful risk-benefit assessment [18]. The questions remain open (1) whether the novel oral agents will replace ASCT and (2) whether we should use them as post-transplant maintenance. For today, this is not a case in countries like Poland where the availability of pathway inhibitors is limited and ASCT remains an important option for poor-risk CLL patients.
